# Supplementary material for: Identification of photoperiod-induced specific miRNAs in the adrenal glands of Sunite sheep (Ovis aries)
Source: Front Vet Sci. 2022 Jul 22;9:888207. doi: 10.3389/fvets.2022.888207 (PMC9354845; doi:10.3389/fvets.2022.888207)
Supplement: Supplementary file 1 [file Data_Sheet_1.ZIP › Supplementary file/miRDeep2 file.zip!/miRDeep2 file/Know_Structure/SPLP42A2/oar-mir-21.pdf]

miRBase precursor : oar-mir-21
Total read count : 240140
oar-miR-21 read count : 240114
remaining reads : 26

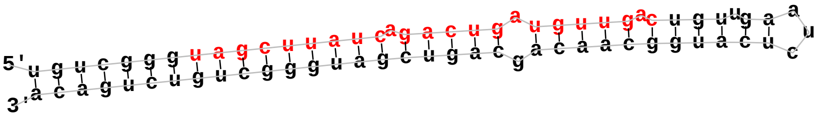

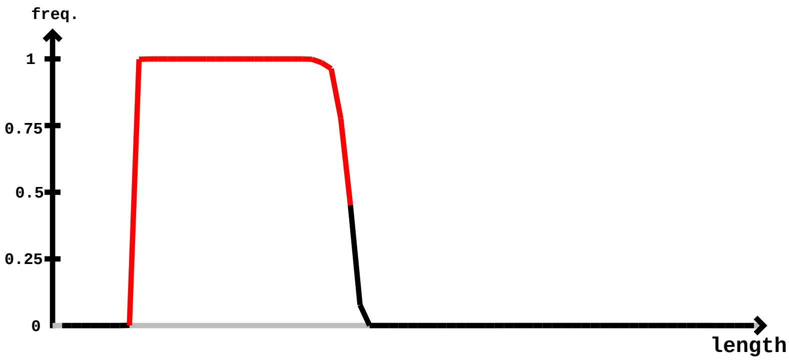


```
        oar-miR-21
5'- ugucggguagcuuaucagacugauguugacuguugaaucucauggcaacagcagucgaugggcugucugaca   -3'    exp
    ((((((((((((((((.(((((.(((((.((((.((...))))))))))).))))))))))))))))))))    reads      mm         sample
    ......guagcuuaucagacugaugu.............................................    6          0          seq
    ......guagcuuaucagacugauguu............................................    7          0          seq
    ......guagcuuaucagacugauguug...........................................    87         0          seq
    ......guagcuuaucagacugauguuga..........................................    68         0          seq
    ......guagcuuaucagacugauguugac.........................................    30         0          seq
    ......guagcuuaucagacugauguugacu........................................    11         0          seq
    .......uUgcuuaucagacugau...............................................    1          1          seq
    .......uagAuuaucagacugau...............................................    1          1          seq
    .......uagcuuaucagacugau...............................................    20         0          seq
    .......uagcuuaucagacugaug..............................................    320        0          seq
    .......uagcuuaucagacugaugu.............................................    2996       0          seq
    .......uagcuuaucagacugauguu............................................    5485       0          seq
    .......uagcuuaucagacugauguug...........................................    44838      0          seq
    .......uagcuuaucagacugauguuga..........................................    77783      0          seq
    .......uagcuuaucagacugauguugac.........................................    89607      0          seq
    .......uagcuuaucagacugauguugacu........................................    18542      0          seq
    .......uagcuuaucagacugauguugacug.......................................    6          0          seq
    .......uagcuuaucagacugauguugacugu......................................    10         0          seq
    .......uagcuuaucagacugauguugacuguu.....................................    11         0          seq
    .......uagcuuaucagacugauguugacuguug....................................    5          0          seq
    .......uagcuuaucagacugauguugacuguugaau.................................    1          0          seq
    ........agcuuaucagacugauguu............................................    6          0          seq
    ........agcuuaucagacugauguug...........................................    40         0          seq
    ........agcuuaucagacugauguuga..........................................    58         0          seq
    ........agcuuaucagacugauguugac.........................................    95         0          seq
    ........agcuuaucagacugauguugacu........................................    29         0          seq
    .........gcuuaucagacugauguug...........................................    11         0          seq
    .........gcuuaucagacugauguuga..........................................    12         0          seq
    .........gcuuaucagacugauguugac.........................................    17         0          seq
    .........gcuuaucagacugauguugacu........................................    5          0          seq
    ..........cuuaucagacugauguu............................................    1          0          seq
    ..........cuuaucagacugauguug...........................................    1          0          seq
    ...........uuaucagacugauguug...........................................    2          0          seq
    ...........uuaucagacugauguugac.........................................    3          0          seq
    .....................................cucauggcaacagcagucgaugggcugu......    1          0          seq
```

oar-miR-21

```
ugucggguagcuuaucagacugauguugacuguugaaucucauggcaacagcagucgaugggcugucugaca
............................................caacagcagucgaugggcug.......    1    0    seq
............................................caacagcagucgaugggcugu......    9    0    seq
............................................caacagcagucgaugggcuguc.....    14   0    seq
```
